# Investigating the paradox of increasing obesity and declining heart disease mortality in the United States: Age-period-cohort model

**DOI:** 10.3389/fcvm.2022.948561

**Published:** 2022-08-17

**Authors:** Bin Yu, Xinguang Chen, Dandan Lu, Hong Yan, Peigang Wang

**Affiliations:** ^1^Department of Epidemiology and Health Statistics, School of Public Health, Wuhan University, Wuhan, China; ^2^Population and Health Research Center, Wuhan University, Wuhan, China; ^3^Department of Surgery, Duke University School of Medicine, Durham, NC, United States; ^4^Deprtment of Epidemiology, University of Florida, Gainesville, FL, United States; ^5^Global Health Institute, Xi’an Jiaotong University, Xi’an, China; ^6^Department of Social Medicine and Health Management, School of Public Health, Wuhan University, Wuhan, China

**Keywords:** heart disease, obesity, cigarette smoking, age-period-cohort (APC) analysis, adjusted rates

## Abstract

**Background:**

Obesity as a risk factor of heart disease (HD) is confirmed through observational, laboratory, and intervention studies. However, it cannot explain why HD declines, but obesity increases in the United States in recent decades. This study attempted to understand this paradox.

**Methods:**

Annual data of national HD mortality (1999–2018) were derived from Wide-Ranging Online Data for Epidemiologic Research, biannual obesity data (1999–2016) from the National Health and Nutrition Examination Survey, and smoking data (1965–1990) were from the National Health Interview Survey. Age-period-cohort method was used to decompose HD mortality into age, period, and cohort effects, and to estimate age-cohort adjusted mortality rates. To explain the paradox, age-cohort adjusted rates were associated with obesity rates to verify the positive obesity-HD relationship, while smoking rates were associated with cohort effects to explain the current declines in HD mortality.

**Results:**

During 1999–2018, the prevalence of obesity increased while the crude HD mortality rate declined for both sex and all races. After controlling for the curvilinear age effect and consistent declining cohort effect, the age-cohort adjusted HD mortality sustained stable in 1999–2007 and increased thereafter. The age-cohort adjusted rate in 1999–2018 (per 100,000) increased from 189.31 to 238.56 for males, 67.23 to 90.28 for females, 115.54 to 157.39 for White, 246.40 to 292.59 for Black, 79.79 to 101.40 for Hispanics, and 49.95 to 62.86 for Asian. The age-cohort adjusted HD mortality rates were positively associated with obesity rates (*r* = 0.68 for males, 0.91 for females, 0.89 for White, and 0.69 for Hispanic, *p* < 0.05), but not significant for Black and Asian. Further, during 1965–1990, the estimated cohort effect showed a decline in HD risk and was positively associated with smoking rates (*r* = 0.98 for both sex, 0.99 for White, and 0.98 for Black, *p* < 0.01).

**Conclusion:**

Study findings reveal potential increase of HD risk and support the positive relationship between obesity and HD risk. Declines in HD mortality in the past two decades are primarily due to tobacco use reduction and this protective effect was entangled in the mortality rates as cohort effect.

## Introduction

Heart and other cardiovascular diseases (CVD) are the second leading cause of death in the United States with over 647,000 deaths per year (1). Effective control and prevention of these diseases require valid data on risk factors, particularly overweight and obese in the present century. Although findings from almost all lines of studies support the role of overweight and obesity as a major risk factor for CVD, particularly heart diseases (HD) mortality (2–5), this conclusion cannot explain the observed remarkable declines in HD mortality in the United States in the past decades. For example, the age-standardized HD mortality rate (per 100,000) in the United States fell by 68.4% from 668.2 in 1969 to 211.1 in 2014 for men, and by 67.6% during the same period from 404.4 to 131.1 for women (6). Meanwhile, the rate of obesity among adults in the United States increased approximately to 200% from 13.4% in 1960–1962 to 40% in 2015–2016 (7).

The remarkable paradox of increasing obesity prevalence and declining HD mortality has long been recognized (8–10); but few empirical studies if any have resolved the issue. This paradox can hardly be attributable to the measurement errors in HD mortality and body weight. Although errors cannot be fully ruled out when HD deaths, height, and weight are measured, their impact on the observed temporal trends would be minor. Additionally, the paradox can hardly be due to error in studies examining the relationship between obesity and risk of HD, since the positive relationship is supported by consistent findings from different types of studies conducted in various settings, including laboratory-based studies (11), observational studies (12), and randomized trials (13). The question remains: where does this paradox originate?

One likely explanation of the obesity-HD mortality paradox could be the use of the crude mortality rate which is computed by division of the number of deaths by population. Temporal trends measured by crude rates are known to be confounded by age; and the problem is solved by using age-standardized rates to describe temporal trends. However, recent studies demonstrate that age-standardized rates can further be confounded by birth cohort, since people in current year were born in different years in the past (14–17). This confounding effect could be large for HD risk in the United States because of great success in controlling the key risk factors, particularly the success of tobacco use in the past before body weight increased as a public health issue (18).

Epidemiologically, the HD risk of a population in a year consists of three time-related and closely intertwined components: age effect, period effect, and cohort effect (14–17, 19, 20). *Age effect* refers to the HD risk associated with chronological ages with higher risk for people of older age, and the risk is largely attributed to the biological aging process. *Period effect* refers to the HD risk associated with changes in key risk factors over time that may exert effect on all age groups, and the risk represents the “true” temporal trend. *Cohort effect* likewise refers to the HD risk associated with changes in the key risk factors over the period when the subjects were born, and the risk represents changes in key risk factors in the past uniquely related to the specific birth cohort (14–17).

With mortality data by age for multiple years, age-period-cohort (APC) analysis, a classic epidemiologic model can disentangle the age, period, and cohort effect intertwined in the data. With the estimated age, period, and cohort effects, age-cohort adjusted rates can be estimated to measure the “true” temporal trends of HD risk. This provides a powerful approach to investigate the obesity-HD risk paradox. However, no reported study in the literature has addressed the HD-obesity paradox using this analytical approach.

In this study, we will investigate the paradox as follows with national data. First, we will determine whether HD risk in the United States increased or declined in the past using age-cohort adjusted rates. Second, if the adjusted rate shows an increase in the risk for HD mortality, we will further associate the adjusted rates with prevalence rates of obesity to confirm the relationship between obesity and HD risk. Third, since the reported declines in HD risk were based on age-standardized rates, a declining cohort effect would be the main explanation for an increasing trend described by age-cohort adjusted rates. Finally, we will associate the estimated cohort effects in the past with prevalence rates of tobacco use in the past to confirm the cohort effect.

## Methods

### Data source

Data regarding the mortality of HDs were derived from the Wide-Ranging Online Data for Epidemiologic Research database, a public domain database sponsored by Center for Disease Prevention and Control (21). Deaths of HDs were those coded as I00-I09, I11, I13, and I20-I51 following the definition of diseases of heart in the International Statistical Classification of Diseases and Related Health Problems, Tenth Revision (ICD-10). Annual data by sex for total population and total deaths of HDs by single year of age from 25 to 84 years from 1999 to 2018 were used to calculate mortality rate (1/100,000) for further analysis.

Annual prevalence rates of obesity during 1999–2018 were used for analysis. These rates were derived from National Health and Nutrition Examination Survey from 1999–2000 through 2017–2018 (22) by linear interpolation. Individuals with a Body Mass Index (BMI) greater than or equal to 30 kg/m^2^ were classified as obesity.

Annual prevalence rates of current cigarette smoker among adults during 1965–1990 were used for analysis. The data were derived from the American Lung Association (23). Current smokers were people who have ever smoked 100 cigarettes in lifetime and also smoked in the past 30 days. Annual smoking rates were interpolated using the original rates in 1965, 1970, 1974, 1980, and 1990, respectively, and were used to investigate the correlation between the cohort effects and the smoking rates.

The study was approved by the Institutional Review Board at the University of Florida. This study is secondary data analysis, and no informed consent was required.

### Measurement for age-period-cohort modeling

#### Outcome –mortality of heart disease

The outcome variable was the mortality rate of HDs, which was estimated as the number of deaths due to HDs divided by the total population. The mortality rate was computed by single year of age by sex in each year from 1999 to 2018.

### Time-related predictors for age-period-cohort modeling

Three time-related predictors were chronological age, time period, and birth cohort. The chronological age was the registered age when the death occurred, and it ranged from 25 to 84 years in the study. Time period was the year when the death was reported, ranging from 1999 to 2018. Birth cohort was computed by subtracting the age from the time period with a range of 1915 to 1993.

### Statistical analysis

The crude mortality rate of HDs was analyzed using APC modeling analysis (19, 20). With an assumption of Poisson distribution, the mortality rates were modeled using the following equation:


(1) $$\log\left(r_{i\,j\,k}\right)=u+\alpha_{i}\,\left(a\,g\,e_{i}\right)+\beta_{j}\,\left(p\,e\,r\,i\,o\,d_{j}\right)+\gamma_{k}\,\left(c\,o\,h\,o\,r\,t_{k}\right)$$

where *r*_*ijk*_ indicates the HD mortality rate, *u* was the intercept or grand mean, α_*i*_ was the effect for age group *i* (*i* = 25, 26, 27, 28, …, 84), β_*j*_ was the effect for period *j* (*j* = 1999, 2000, 2001, …, 2018), and γ_*k*_ was the effect for birth cohort *k* (*k* = 1915, 1916, …, 1993).

The parameters in the model were estimated using the Intrinsic Estimator (13) in the statistical software Stata, version 15.0 with the package “apc” (StataCorp). The estimated age, period, and cohort effects were plotted by sex to show the time trend.

The age-adjusted rate was directly derived from the CDC WONDER. Based on the modeling results, the age-cohort adjusted mortality rate of HD was computed using the following formula (14):


(2) $$\text{Age-cohort adjusted rate}=\frac{e^{u+\beta_{j}+\alpha_{m\,e\,d\,i\,a\,n}+\gamma_{m\,e\,d\,i\,a\,n}}}{1+e^{u+\beta_{j}+\alpha_{m\,e\,d\,i\,a\,n}+\gamma_{m\,e\,d\,i\,a\,n}}}$$

where *u* was the intercept, β_*j*_ was the period effect (*j* = 1999, 2000, 2001…2017), α_*median*_ was the median of age effects, and γ_*median*_ was the median of cohort effects. These effects were estimated through Equation 1. To better visualize the differences of crude rate, age-adjusted rate, and age-cohort adjusted rate, the three rates were plotted together by sex and race.

Finally, Pearson correlation was used to correlate estimated smoking rates with estimated cohort effects, as well as obesity rates with crude, age-adjusted and age-cohort adjusted HD mortality rates. Type I error was set at *p* < 0.05 (two-sided) in statistical inference.

## Result

### Time trends in heart disease mortality and obesity

**FIGURE 1:**
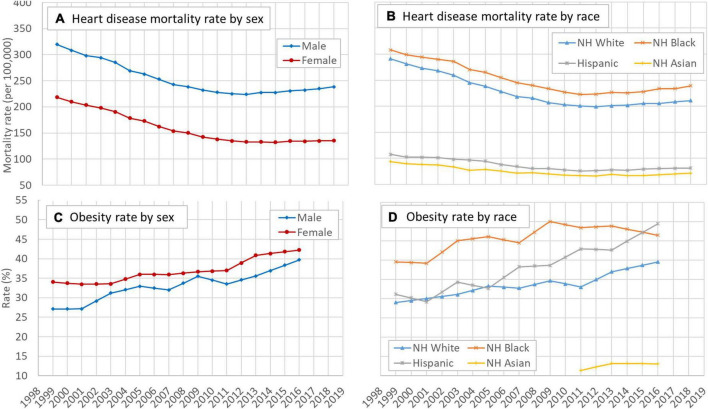
Time trend of mortality rates of HD and the obesity rate in the US, 25–84 years, 1999–2018, by sex and race. **(A)** Temporal trend of heart disease mortality rate by sex, **(B)** temporal trend of heart disease mortality rate by race, **(C)** temporal trend of obesity rate by sex, and **(D)** temporal trend of obesity rate by race. HD: Heart disease; NH: Non-Hispanic. Data source: CDC WONDER (https://wonder.cdc.gov/). Data about the obesity rate were derived from the NHANES.

Figures 1A,B indicate that despite obvious differences in magnitude, overall the crude HD mortality rate (1/100,000) showed a declining trend during the study period for different sex and racial groups although the trend slowed down in recent years, particularly for males and Whites. More details of the crude HD mortality rates are presented in Supplementary Table 1.

Likewise, Figures 1C,D indicate that overall the prevalence rates of obesity during 1999–2018 increased for sex and racial groups. The speed of increase was higher for males than for females and the speed was the highest among Hispanics compared to other racial groups.

### Age, period, and cohort effects of mortality of heart diseases

**FIGURE 2:**
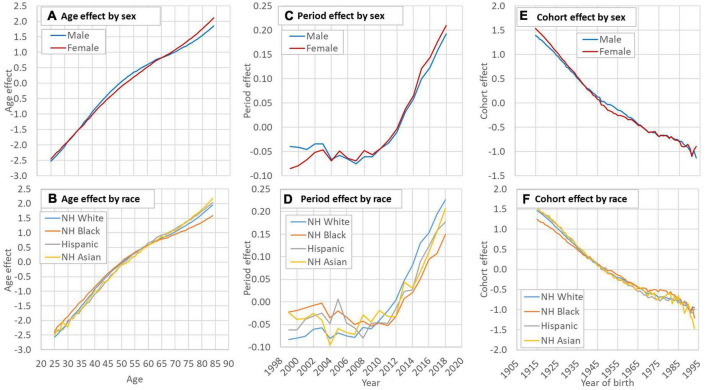
Decomposed age **(A,B)**, period **(C,D)** and cohort **(E,F)** effect of HD mortality in the US by sex and race. HD: Heart disease; NH: Non-Hispanic. Data source: CDC WONDER (https://wonder.cdc.gov/). Data of the smoking rate among Hispanic and Asian were unavailable before 1993.

Figures 2A,B indicate a curvilinear net effect of age on HD risk, and the pattern was similar across sex and racial groups. Figures 2C,D depict the net effect of time period on HD risk from 1999 to 2018 by sex and racial groups, reflecting underlying time trend of HD risk in the United States across different groups. Overall, the risk increased with an obvious acceleration since 2007 with risk increased quickest for Whites and slowest for Blacks. The estimated cohort effect in Figures 2E,F showed a consistent declining trend in HD risk during 1915–1993 with small differences across sex and racial groups.

### Comparison of crude and adjusted rates of heart disease mortality

**FIGURE 3:**
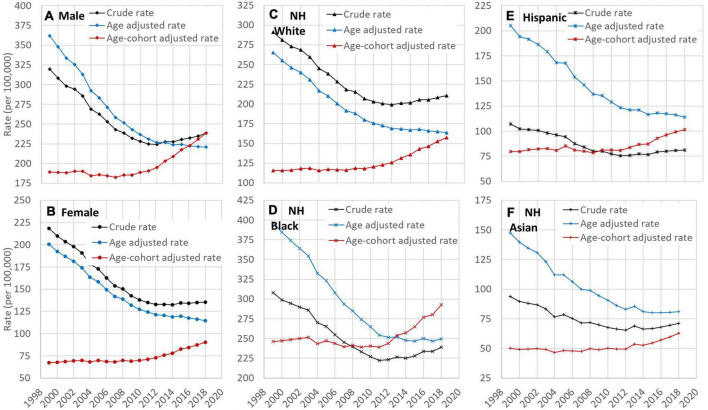
Time trend of crude, age-adjusted, and age and cohort-adjusted rates of HD mortality among US adults, 25–84 years old, 1999–2018 by sex **(A,B)** and race **(C–F)**. HD: Heart disease; NH: Non-Hispanic. Data source: CDC WONDER (https://wonder.cdc.gov/).

Crude, age-adjusted, and age-cohort adjusted rate of HD mortality by sex and racial groups are presented in Figures 3A–F. In all groups, both the crude rates and the age-adjusted rates showed a decline trend while the age-cohort adjusted rates showed an increasing trend during the study period. Results in Figure 3 further indicate that the time trend in HD risk by crude death rates was confounded primarily by the birth cohort.

### Correlation of obesity crude rate and adjusted rates

Results in Table 1 indicate that the obesity rate was positively associated with the age-cohort adjusted rates, supporting not only validity of the adjusted rates but also the conclusion that obesity is a risk factor for HD. This result was consistent for both sex with estimate *r* = 0.68 (*p* < 0.05) for male and *r* = 0.91 (*p* < 0.001) for female, and for White (*r* = 0.89, *p* < 0.001) and Hispanic (*r* = 0.69, *p* < 0.05).

**TABLE 1:** Correlations (1) between obesity rates and crude rate, age adjusted, and age-cohort adjusted HD mortality rates (1999–2018) and (2) between cohort effects and smoking rates (1965–1990), by sex and racial groups.

Variables	Crude rate	Age adjusted rate	Age-cohort adjusted rate	Cohort effect
**Rate of obesity**				
*By sex*				
Male	−0.88**	−0.92**	0.68*	
Female	−0.83**	−0.86**	0.91**	
*By racial groups*				
NH White	−0.83**	−0.88**	0.89**	
NH Black	−0.91**	−0.90**	−0.02	
Hispanic	−0.88**	−0.94**	0.69*	
Asian	0.49	−0.63	0.78	
**Rate of smoking**				
*By sex*				
Male				0.98**
Female				0.98**
*By racial groups*				
NH White				0.99**
NH Black				0.98**
Hispanic				NA
Asian				NA

^1^HD: Heart disease; NH: Non-Hispanic.

^2^Student t-test of correlation coefficient r: *: p < 0.05, **: p < 0.001.

^3^No smoking data were available for Hispanic and Asian during 1965-1990.

### Correlation between cohort effects and smoking rates

Results in Table 1 also show that the correlation coefficient between smoking rates and cohort effects was 0.98 (*p* < 0.01) for both sex, and 0.99 (*p* < 0.01) for White and 0.98 (*P* < 0.01) for Black.

## Discussion

In this study, we successfully extracted information from annual mortality data to investigate the paradox of declining HD mortality along with increasing obesity in United States with national data. According to our analysis, the paradox is primarily due to the incorrect description of a decline trend in HD mortality in the United States using crude or age-standardized rates. These rates are confounded by a strong and sustained declining cohort effect; they are not valid to characterize temporal trends in HD risk. To our knowledge, this is the first empirical study to generate data interpreting the obesity-HD mortality paradox. The paradox was recognized more than a decade ago and reduces our beliefs on the role of weight control for HD prevention (8). In addition to dissecting this paradox, findings of the study add new information deepening our understanding of the relationship between obesity and HD risk at the population level.

Previously documented data concluded that the risk of HD mortality has been declining in industrialized countries, including the United States (6, 9). Therapeutic advancement was estimated to account for approximately 50% of the decline (24, 25). Findings of this study challenge this conclusion. Instead of declines, the age-cohort adjusted rates of HD increased since 1999. Furthermore, the increase accelerated in recent years for both sexes and all racial/ethnic subgroups. The declining trends indicated by crude and age-adjusted rates are confounded by the birth cohort. The observed declines in HD mortality in recent years are mainly due to cohort effects. The positive relation between the estimated cohort effects and smoking rates provides data further supporting the cohort effect from tobacco control that contributes to the recent declines in HD risk (9).

Previous studies also revealed that the declining trend in HD mortality decelerated, and even showed early signs of reversal in some population subgroups, despite continuous advancements in diagnosis and treatment (9, 26–31). However, these studies did not examine the confounding impact of cohort effect on the temporal trend. Thus, findings from these studies are inadequate to address the obesity-HD mortality paradox and to inform evidence-based weight control programs for HD prevention and mortality reduction.

Findings of this study add new evidence supporting the need for weight-control. Overweight and obese are related to increased HD mortality rates and higher blood pressure in the United States, and individuals with extra bodyweight and hypertension are at increased risk for HD (2, 3). Of the hypertensive individuals, nearly three-quarters are either overweight or obese (4, 5). There is no doubt about the threat of overweight and obesity, and decision-makers, preventive and clinical professionals and the public alike should be aware of the increasing threat and to support body weight control as an important prevention measure.

One unique and significant contribution of this study is explanation of the obesity-HD mortality paradox. In addition to detecting the increasing trend in HD mortality, there is a positive relation between obesity rate and age-cohort adjusted HD mortality rates. The relationship would be negative if age and cohort are not justified. Findings of this analysis confirm the role of bodyweight as an important predictor of HD, and potentially CVD in general as reported in previous studies (12, 32, 33).

Additionally, an overall decline of cohort effect was observed which was significantly correlated with smoking rates. Previous studies indicated that nearly half of the CVD mortality decline can be attributed to the prevention of risk factors, including cigarette smoking (9). Our findings provide additional data supporting the effect of tobacco controls in reducing risk of HD (18). Further, the decline of cohort effect was slowing down indicating the diminishing of the protective effect of historical prevention programs targeting on risk factors. As the cohort effect diminished, the risk of HD started to increase in response to increases in obesity over time.

Finally, we must point out that it is highly misleading to use crude rates or even age-standardized rates to describe time trends of chronic disease mortality. To date, few studies in the literature detected the increasing trend in HD mortality although age-standardized rates were often used, including studies based on the clinical data and population-based survey data. Different from infectious diseases, HD and other CVD alike are chronic in nature; it takes time to complete the process from disease onset to clinical diagnosis, treatment and to the outcome of death. Effect of various influential factors is intertwined with each other. As described in APC modeling, much of which shows as a cohort effect hidden in current data (14, 15, 34, 35).

## Limitations

The study has limitations. First, the analysis was conducted with aggregated data. Effect related to advancement in HD diagnosis and treatment and factors at the individual level cannot be examined. Second, it would be ideal to also use age-cohort adjusted rates of smoking and obesity; but annual data by age for the two measures are not available. The smoking-HD mortality has been well established; regarding the obesity-HD, we believe that using adjusted rates would not change those results qualitatively since obesity has been progressively increasing. However, we recommend using caution while reviewing this part of the results. Last, a full investigation of all factors related to the declining cohort effect and increasing period effect on HD mortality is likely but beyond the scope of the study. We will consider this issue in our future studies.

## Conclusion

HD risk in the United States increases rather than declines since 1999, and the increased risk is associated with obesity. The obesity-HD paradox is primarily due to confounding effect from birth cohorts that are related with reductions in tobacco use since 1965. Findings of this study not only provide answers to the two-decade long paradox regarding bodyweight and HD risk but also demonstrate increase in HD risk in the Unied States and the significance of bodyweight control in CVD prevention and control. Last, age-cohort adjusted rates provide a measure more valid than crude or age-adjusted rates to describe trend of HD risk. We highly recommend the use of this measure in future studies.

## Data availability statement

Publicly available datasets were analyzed in this study. This data can be found here: https://wonder.cdc.gov/Welcome.html.

## Ethics statement

This study was reviewed and approved by the Institutional Review Board at the University of Florida. This study is second data analysis, and no informed consent was required.

## Author contributions

BY contributed to the study design, data collection, analysis and interpretation, and writing the first draft. XC contributed to the study design and manuscript review. DL, HY, and PW contributed to the manuscript review. All authors contributed to the article and approved the submitted version.
