# Supplementary material for: Investigating the paradox of increasing obesity and declining heart disease mortality in the United States: Age-period-cohort model
Source: Front Cardiovasc Med. 2022 Aug 17;9:948561. doi: 10.3389/fcvm.2022.948561 (PMC9429831; doi:10.3389/fcvm.2022.948561)
Supplement: Supplementary file 1 [file Table_1.DOCX]

Table S1. The crude mortality (per 100,000) of heart diseases of the study sample, overall, by sex and by race/ethnicity

| **Year** | **Total** | **Male** | **Female** | **White** | **Black** | **Hispanic** | **Asian** |
| --- | --- | --- | --- | --- | --- | --- | --- |
| 1999 | 267.4 | 319.8 | 218.5 | 291.2 | 307.9 | 107.2 | 93.7 |
| 2000 | 257.4 | 308.2 | 209.9 | 281.3 | 299.2 | 102.1 | 89.7 |
| 2001 | 249.3 | 298.0 | 203.6 | 272.9 | 294.5 | 101.7 | 88.0 |
| 2002 | 244.6 | 294.1 | 198.2 | 268.5 | 289.9 | 100.9 | 86.8 |
| 2003 | 236.5 | 285.4 | 190.6 | 259.8 | 285.9 | 98.0 | 83.2 |
| 2004 | 222.3 | 269.0 | 178.4 | 245.0 | 270.2 | 93.4 | 76.7 |
| 2005 | 216.5 | 262.8 | 173.0 | 238.5 | 265.3 | 94.3 | 78.7 |
| 2006 | 206.4 | 253.0 | 162.5 | 228.2 | 254.9 | 87.6 | 75.4 |
| 2007 | 197.0 | 242.8 | 153.8 | 218.5 | 245.1 | 84.1 | 71.6 |
| 2008 | 193.2 | 238.7 | 150.3 | 215.4 | 239.8 | 80.0 | 72.0 |
| 2009 | 186.0 | 232.1 | 142.5 | 206.8 | 233.6 | 80.1 | 69.9 |
| 2010 | 181.7 | 228.0 | 138.0 | 203.0 | 227.0 | 77.4 | 67.7 |
| 2011 | 178.7 | 225.0 | 134.9 | 200.7 | 222.5 | 75.7 | 66.5 |
| 2012 | 177.3 | 224.2 | 132.9 | 198.9 | 223.2 | 75.9 | 65.5 |
| 2013 | 179.0 | 227.4 | 133.0 | 200.9 | 226.5 | 77.3 | 69.0 |
| 2014 | 178.7 | 227.6 | 132.3 | 201.7 | 225.4 | 76.5 | 66.4 |
| 2015 | 181.5 | 230.6 | 134.7 | 205.2 | 227.9 | 79.2 | 66.8 |
| 2016 | 182.1 | 232.4 | 134.3 | 205.5 | 233.9 | 80.0 | 68.0 |
| 2017 | 183.6 | 234.8 | 134.9 | 208.5 | 233.7 | 80.7 | 69.4 |
| 2018 | 185.6 | 238.5 | 135.2 | 210.8 | 239.1 | 81.0 | 71.1 |
